# Accidental spinal cord injury during spinal anesthesia: A report

**DOI:** 10.4103/0972-2327.74200

**Published:** 2010

**Authors:** M. Netravathi, A. B. Taly, S. Sinha, P. S. Bindu, G. Goel

**Affiliations:** Department of Neurology, National Institute of Mental Health & Neurosciences, Bangalore, India; 1 Department of Neuroimaging and Interventional Radiology, National Institute of Mental Health & Neurosciences, Bangalore, India

## Introduction

We describe the clinical and radiologic observations of a patient who developed neurologic deficits following spinal anesthesia (SA) for caesarian section.

**Figure 1:**
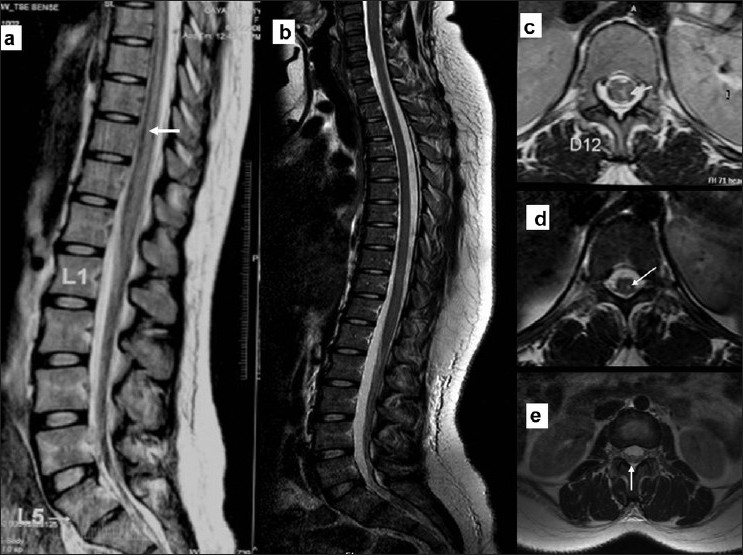
MRI immediately following neurologic deficits after spinal anesthesia and at 3 months follow-up. (a) T2-weighted (T2W) sagittal image of the lower dorsal and lumbosacral cord showing hyperintensities with cord edema at presentation; (b) sagittal T2W image at follow-up after 3 months showing decreased signal changes and edema; (c) T2W axial image of the lumbar cord demonstrating hyperintensity with cord edema at presentation; (d) T2W axial image of the follow-up scan after 3 months with hyperintensity within the cord with reduced cord edema; and (e) T2W axial image of the lumbar cord revealing clumped nerve roots suggestive of arachnoiditis 3 months after presentation.

A 21-year-old primipara underwent SA in the right lateral position for an emergency caesarian section. The injection was initially at L_1_–L_2_ site using a 25-gauge spinal needle. Since there was no free flow of cerebrospinal fluid, second attempt was made at T_12_–L_1_ interspace. A bolus dose of 1.6 mL 0.5% bupivacaine was injected into the subarachnoid space. The patient experienced radiating pain down the left lower extremity during the procedure. She did not report this immediately. Hence, it could not be ascertained whether the radiating pain was during the needle prick or during the injection of local anesthetic. The operation was carried out in the supine position and lasted for 45 min. The very next day she noted persistent numbness and weakness of her left lower limb with normal bladder and bowel sensations. She was subsequently referred to us. At presentation, the vitals, general physical and systemic examination were unremarkable. She had moderate weakness of the left lower limb: proximal–3/5 and distal–2/5 [medical research council (MRC) grade]. All the muscle stretch reflexes in the lower limbs were absent except right knee that was easily elicitable. There was dissociative sensory loss on the left side of the abdomen and lower limb below T8 with absent pain and temperature sensations and preserved touch/kinesthetic sensations. Routine investigations were normal. Magnetic resonance imaging (MRI) of the spine that was carried out a week later revealed lower thoracic cord expansion with extensive signal changes extending from T_7_to T_12_, which was isointense on T1-weighted (T1W) images and hyperintense on T2-weighted (T2W) images [Figure 1a, c] without contrast enhancement. A possibility of spinal anesthesia-related traumatic spinal cord injury was considered. She received intravenous methylprednisolone (1 g/day) for 5 days followed by a short course of oral steroids. The patient improved gradually and 3 months later, she was ambulant with minimal weakness of the ankle dorsiflexors and plantar flexors (MRC: 3/5), but the stretch reflexes remained absent. The dissociative sensory loss also persisted. Repeat MRI (spine) showed T1W hypointense and T2W hyperintense lesion across T_6_–T_8_without any contrast enhancement and decreased cord size, suggestive of residual gliosis as sequelae to the previous trauma [Figures 1b, d]. There was clumping of adjacent nerve roots suggestive of arachnoiditis [Figure 1e].

The neurologic deficits following SA could be due to direct trauma by the needle or spinal cord ischemia due to either hypotension or vasoconstrictors used along with the local anesthetic agents.[1–4] Severe neurologic complications resulting from spinal anesthesia are rarely reported.[5,6] Our patient developed motor and sensory impairment in left lower limb after SA with corresponding changes in spinal MRI. The mechanisms responsible for the early onset of neurologic symptoms could be due to direct needle injury of the spinal cord and injection of the local anesthetic into the cord parenchyma, leading to immune-mediated changes and myelitis.

Accidental puncture of the underlying cord elicits severe pain and reflex movement in conscious patients and one should withdraw the needle immediately. Permanent injury follows if this warning is ignored and local anesthetic is injected into the cord. The value of high-dose methylprednisolone is unknown but might be considered. Prognosis of these patients is gradual motor recovery over a period of days to months due to the resolution of edema. Unfortunately, the spinothalamic impairment may persist and in few cases leads to severe spontaneous pain over the affected segment.

This report reemphasizes that lumbar puncture is a “blind” procedure and one should follow the standard procedure and all patients should undergo the recommended checklist.
